# Thrombocytopenia and Acute Renal Failure in Puumala Hantavirus Infections

**DOI:** 10.3201/eid1008.031069

**Published:** 2004-08

**Authors:** Franz Maximilian Rasche, Boris Uhel, Rainer Ulrich, Detlev H. Krüger, Wolfram Karges, David Czock, Walter Hampl, Frieder Keller, Helga Meisel, Lutz von Müller

**Affiliations:** * University Hospital Ulm, Ulm, Germany,; † Charité, Berlin, Germany

**Keywords:** acute renal failure, hemodialysis, hantavirus, nephropathia epidemica, Puumala virus, thrombocytopenia, research

## Abstract

Nephropathia epidemica, caused by Puumala virus (PUUV) infection, is a form of hemorrhagic fever with renal syndrome of variable severity. Early prognostic markers for the severity of renal failure have not been established. We evaluated clinical and laboratory parameters of 15 consecutive patients with acute PUUV infection, which is endemic in the Alb-Danube region, South Germany. Severe renal failure (serum creatinine >620 µmol/L) was observed in seven patients; four required hemodialysis treatment. Low platelet count (<60 x 10^9^/L), but not leukocyte count, C-reactive protein, or other parameters obtained at the initial evaluation, was significantly associated with subsequent severe renal failure (p = 0.004). Maximum serum creatinine was preceded by platelet count nadirs by a median of 4 days. Thrombocytopenia <60 x 10^9^/L appears predictive of a severe course of acute renal failure in nephropathia epidemica, with potential value for risk-adapted clinical disease management.

## Abstract 2

Low platelet counts are a novel predictive marker suitable for risk-adapted patient management.

Worldwide, approximately 60,000–150,000 patients per year are hospitalized with hantavirus infections (1,2). *Hantavirus* spp. (*Bunyaviridae* family) are transmitted to humans by inhalation of aerosolized excreta of persistently infected rodents (3,4). Puumala virus (PUUV) is a representative of this genus responsible for most cases of hantavirus infections in northern Europe (1,5). The red bank vole (*Clethrionomys glareolus*) is the natural host reservoir of PUUV. Nephropathia epidemica attributable to acute PUUV infection is a mild form of hemorrhagic fever with renal syndrome (1,4–6). Endemic seasonal outbreaks of PUUV infections are common in Scandinavia, but outbreaks in central Europe are restricted to distinct regions, e.g., in South Germany (2,4,7).

Nephropathia epidemica is characterized by acute fever, headache, nausea, vomiting, myalgia, abdominal and loin pain, mild hepatitis and pancreatitis, and interstitial nephritis with acute renal failure. Hemodialysis is required in 10% to 30% of hospitalized patients with acute PUUV infections (8). A complete recovery of renal function is regularly achieved after several weeks (1,9). Acute renal failure in nephropathia epidemica is frequently accompanied by thrombocytopenia, elevated leukocyte count, proteinuria, hematuria, and low serum calcium (9–11), but early prognostic markers have not yet been established to identify patients at high risk for a severe course of acute renal impairment. We evaluated clinical and laboratory parameters that could predict the severity of acute renal failure suitable for risk-adapted disease management in patients with nephropathia epidemica.

## Patients and Methods

From 1998 to 2001, all consecutive patients with nephropathia epidemica and serologically confirmed hantavirus infection were studied. Patients were admitted by physicians and regional hospitals to the nephrology division of the University Hospital of Ulm, a center of nephrology and infectious diseases in South Germany with a patient base of 100 km (radius) on both sides of the River Danube. Hemodialysis was started in patients with severe symptoms of uremia (serum creatinine >620 µmol/L, serum urea nitrogen >150 mg/dL, serum potassium >6.0 mmol/L, oliguria <500 mL/day, or progressive body weight increase with edema). After discharge, a follow-up examination of all patients was conducted in our outpatient clinic. Blood pressure, leukocyte count, hemoglobin, platelet count, prothrombin time, activated partial thromboplastin time, C-reactive protein, serum electrolytes, alanine aminotransferase (ALT), serum protein, serum creatinine, urea, nitrogen, proteinuria, and microscopic qualitative and quantitative (Addis count) urine analysis were evaluated. Clinical and laboratory data obtained before admission were also evaluated. The study protocol was approved by the Ethics Committee, University of Ulm, and written informed consent was obtained from all patients. Studies were conducted in accordance with the Declaration of Helsinki.

In all patients, acute hantavirus infection was serologically diagnosed by PUUV-specific immunoglobulin (Ig) G enzyme immunoassay (EIA) (Progen, Heidelberg, Germany). In addition, Hantaan virus (HTNV)-, PUUV- and Dobrava virus (DOBV)-specific IgM and IgG antibodies were detected by in-house monoclonal antibody–capture or µ-capture EIAs (Charité, Berlin), as previously described (12); by immunofluorescence assays (IFA) using HTNV-, PUUV-, and DOBV-infected Vero E6 cells; or by an immunoblot (IB) using recombinant hantavirus antigens (Mikrogen, Martinsried, Germany). In nine patients, hantavirus serotyping was performed by chemiluminescence focus reduction neutralization assays (c-FRNTs), as described recently (13).

Nonparametric tests were used for statistical analysis (Fisher exact test, Mann-Whitney U test), and significance was set at a level of p < 0.05. No adjustment for multiple comparisons was made, and results were interpreted in an exploratory manner. All statistical analyses were conducted with SPSS 8.0 software package (SPSS Inc., Chicago, IL). If not indicated otherwise, data are given as median values with range (minimum to maximum).

## Results

**Figure 1:**
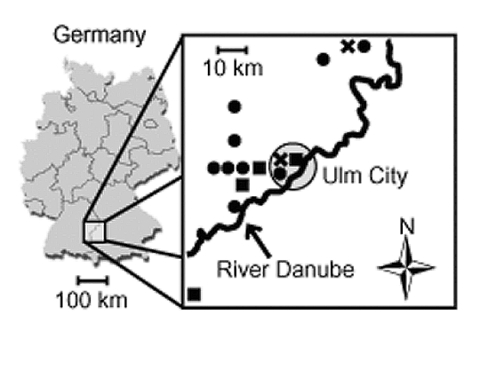
Residence of 15 study patients with hantavirus infection and nephropathia epidemica, according to year of diagnosis (X, November–December 1998; black circle, January–May 2000; black square, September–November 2001). Note the River Danube as a potential natural barrier of Puumala virus hantavirus infection. Shaded circle indicates Ulm city.

Fifteen patients (mean age 37 ± 8 years; male:female ratio 12:3) with nephropathia epidemica were treated at the University Hospital of Ulm from January 1998 to December 2001 (Table 1). PUUV infections varied in different years by number of patients and seasons with a high incidence (Figure 1: November to December 1998: 2 patients; 1999 none; January to May 2000: 9 patients; September to November 2001: 4 patients). All but one patient lived on the north side of the River Danube. No patient was working in agriculture, forestry, or other professions considered at high risk for contact with infected rodent excreta. Acute or chronic use of nonsteroidal antiinflammatory drugs was denied.

**Table 1:** Characteristics of 15 patients with acute PUUV infection associated with mild or severe acute renal failure^a,b^

	Mild acute renal failure (n = 8)	Severe acute renal failure (n = 7)	p value
Age (y)	38 (25–53)	35 (22–53)	n.s.^c,d^
Sex (male:female ratio)	5:3	7:0	n.s.^e^
Fever (>38.5°C)	8	7	n.s.^e^
Abdominal or loin pain	7	5	n.s.^e^
Fatigue	4	6	n.s.^e^
Myalgia	5	4	n.s.^e^
Hepatitis (ALT >20 U/mL)	4	5	n.s.^e^
Headache	4	3	n.s.^e^
Nausea/vomiting	1	5	0.041^e^
Conjunctival bleeding	1	2	n.s.^e^
Purpura	0	2	n.s.^e^
Highest C-reactive protein (mg/L)	50 (27–112)	90 (7–122)	n.s.^d^
Lowest platelet count (x 10^9^/L)^b^	113 (26–250)	34 (18–122)	0.016^d^
Highest leukocyte count (x 10^9^/L)	9.9 (8.0–15.3)	15.1 (12.0–22.7)	0.029^d^
Lowest serum calcium (mmol/L)	2.22 (1.85–2.27)	2.02 (1.98–2.21)	0.029^d^
Hematuria (cells/min)	10.2 (2.6–27)	76.4 (21.2–129.0)	0.009^d^
Leukocyturia (cells/min)	16.2 (10.4–22)	41.0 (17.5–191.0)	0.0017^d^
Proteinuria (>1.5 g/day)	2	4	n.s.^e^
Tubular cell casts	1	5	0.001^e^

^a^PUUV, Puumala virus; mild and severe acute renal failure was defined as serum creatinine <620 µmol/L and >620 µmol/L, respectively; ALT, alanine aminotransferase. ^b^Of all parameters, only thrombocytopenia predicted subsequent severe renal failure. Medians (range) and actual number of patients are given. ^c^n.s., not significant. ^d^Mann-Whitney U test. ^e^Fisher exact test.

Patients were admitted to the hospital 5 days (range 2–10 days) after acute onset of clinical symptoms, but the first laboratory examination had already been performed 3 days (median, range 1–9 days) after onset of clinical symptoms by general practitioners. Prominent clinical and laboratory findings of acute nephropathia epidemica were fever (100%), abdominal or loin pain (80%), fatigue (67%), myalgia (60%), hepatitis (60%), headache (47%), nausea (40%), and conjunctival bleeding (20%) (Table 1). Dyspnea and pulmonary infiltrations were not observed on x-ray images. Ultrasound examination showed intact kidney forms in all patients.

PUUV-reactive IgG and IgM antibodies were detected in all patients (Table 2). EIA-positive results were confirmed by at least one independent test (in-house EIA, IFA, IB). During the acute phase of PUUV infection, cross-reactive neutralizing antibodies to other hantavirus serotypes (HTNV, Tula virus) were detected in two patients (c-FRNT), but PUUV infection was confirmed in the convalescent-phase infection by high endpoint titers for PUUV antibodies compared to other hantavirus serotypes. Because of the limited quantity of serum specimens, the complete array of virologic tests was not performed in all samples, but available serologic data demonstrated acute PUUV infections in all patients (Table 2).

**Table 2:** Virologic results of 15 patients with acute PUUV infection^a^

Patient	IgG-EIA (Progen) index	IgG titers			IgM titers	c-FRNT titers				
	PUUV	PUUV	HTNV	DOBV	PUUV	PUUV	HTNV	DOBV	SEOV	TULV
1	9	6,400	Neg	Neg	3,200	640	<40	160	<40	160
2	2.1	2,560	640	–	2,560	–	–	–	–	–
3	2.2	6,400	400	400	1,600	2,560	<40	<40	<40	40
4	3.3	51,200	6,400	400	12,800	640	160	40	<40	160
5	3.8	51,200	3,200	Neg	Pos^b^	2,560	160	40	<40	40
6	3.1	6,400	800	Neg	6,400	2,560	640	40	<40	160
7	3.1	12,800	800	Neg	1,280	640	<40	<40	–	40
8	4.3	12,800	3,200	800	3,200	2,560	160	40	<40	160
9	1.8	Pos^c^	–	–	Pos^c^	–	–	–	–	–
10	2.9	12,800	3,200	3,200	400	2,560	160	40	<40	160
11	7.1	12,800	1,600	400	1,600	–	–	–	–	–
12	2.9	Pos^c^	–	–	Pos^c^	–	–	–	–	–
13	4.9	51,200	400	Neg	3,200	–	–	–	–	
14	2.5	Pos^c^	–	–	Pos^c^	–	–	–	–	–
15	3.3	12,800	1,600	400	1,600	2,560	40	<40	40	40

^a^PUUV, Puumala virus; Ig, immunoglobulin; EIA, enzyme immunoassay; c-FRNT, chemiluminescence focus reduction neutralization assays; HTNV, Hantaan virus; DOBV, Dobrava virus; SEOV, Seoul virus; TULV, Tula virus; –, not available. ^b^IgM-EIA (Progen, Heidelberg, Germany). ^c^Immunoblot (Mikrogene, Martinsried, Germany).

In all patients, a combination of low platelet count (<150 x 10^9^/L), elevated serum creatinine (>150 µmol/L), or elevated C-reactive protein (>10 mg/L) was detected. In seven patients (47%), severe acute renal impairment with a serum creatinine >620 µmol/L developed; four (27%) of these patients required hemodialysis as an acute intervention for 3 days (range 2–9 days) because of symptoms of uremia. During hospital stay, polyuria (>3 L/day) was present in only three patients (20%), and no patient became anuric (<500 mL/day). Serum protein was >60 g/L in all patients, plasma coagulation parameters were not altered, and mild bleeding signs (subconjunctival bleeding, petechia) were present in only three patients with a platelet count <60 x 10^9^/L. Patients remained in the hospital for 10 days (range 5–19 days), and follow-up examination was conducted at our outpatient clinic for 5 months (range 0.2–12.5 months).

We found that lower platelet count, higher leukocyte count, the presence of tubular cell casts in urine, quantitative hematuria and leukocyturia (Addis count), and nausea were significantly associated with severe acute renal failure (serum creatinine >620 µmol/L) (Table 1). In contrast, age, sex, blood pressure, fever, abdominal or loin pain, myalgia, headaches, fatigue, bleeding signs, hemoglobin, prothrombin time, activated partial thromboplastin time, C-reactive protein, sodium, potassium, ALT, serum protein, and proteinuria were not associated with serum creatinine >620 µmol/L (Table 1).

**Figure 2:**
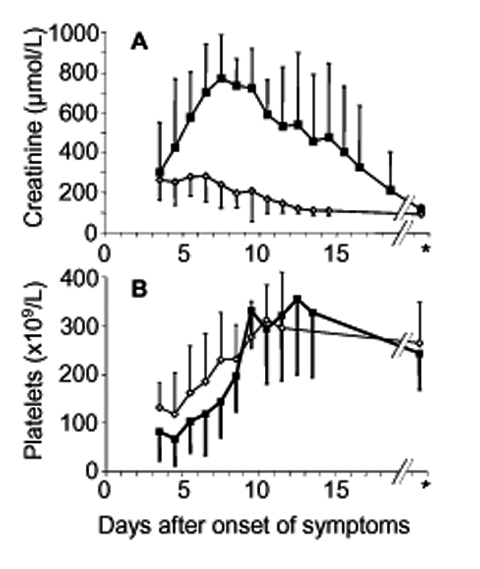
Course of serum creatinine (A) and platelet count (B) in patients with mild (diamond, serum creatinine <620 µmol/L, n = 8) or severe acute renal failure (black square, serum creatinine >620 µmol/L, n = 7). Mean values and SD are shown. *Denotes evaluation at end of followup (median, 5 months).

The platelet count nadir was observed 4 days (range 1–6 days) after the acute onset of clinical symptoms. Seven days after initial symptoms (range 3–12 days), platelet counts had already returned to normal in all patients, with values >150 x 10^9^/L (Figure 2). Maximum serum creatinine values were observed 7 days (range 3–14 days) after onset of disease, and renal function returned to normal within 14 days (range 7–38 days) after onset of symptoms, as defined by serum creatinine <150 µmol/L.

In patients with severe renal failure, platelet count nadirs preceded maximum serum creatinine values by 4 days (range 2–10 days). In patients with a mild or severe course of acute renal failure, the platelet count was different at initial evaluation (p = 0.04), but serum creatinine values at initial evaluation did not differ (p = 0.15). We analyzed the potential prognostic value of low (<60 x 10^9^/L) and moderately decreased (>60 x 10^9^/L) platelet count for the severity of subsequent renal failure. This value represented the median of all patients at first evaluation, which generated two equally sized patient cohorts.

**Figure 3:**
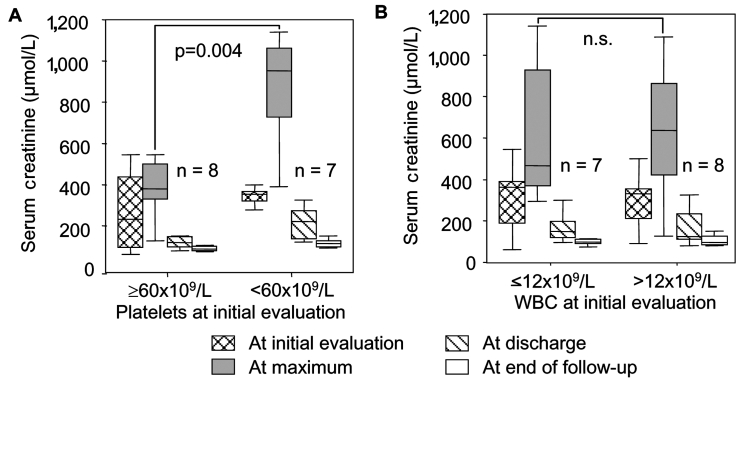
Temporal course of serum creatinine in patients with hantavirus infection, stratified according to (A) platelet count and (B) leukocyte count at initial evaluation. Platelet count, but not leukocyte count, is a significant predictor of subsequent renal failure (p = 0.004, Mann-Whitney). Box plots with median, interquartile range, minimum and maximum values are shown. n.s., not significant; WBC, leukocyte count.

The time interval between initial clinical symptoms and first laboratory evaluation did not differ in the two groups (2 days, range 0–6 days). A platelet count of <60 x 10^9^/L preceded severe renal failure in six of seven patients, and all patients with hemodialysis had a platelet count <60 x 10^9^/L in the early phases of infection. Acute severe renal impairment (sensitivity 0.86, specificity 0.88, positive predictive value 0.86) developed in one of eight patients with a platelet count >60 x 10^9^/L. The initial serum creatinine value was not different in the two groups (p = 0.54, Mann-Whitney U test), but the maximum value was significantly higher in patients with a platelet count of <60 x 10^9^/L during the early phase of infection (Figure 3A). Although associated with a severe course of nephropathia epidemica (Table 1), an increased leukocyte count at first examination was not predictive of the severity of subsequent renal failure (Figure 3B).

## Discussion

Early prognostic parameters for the course of renal impairment might be beneficial for risk-adapted disease management of nephropathia epidemica, but they have not been established to date (1,14). Thrombocytopenia is a well-known transient symptom of this condition (15), and we found that severe thrombocytopenia (<60 x 10^9^/L) was a prognostic parameter predictive for consecutive severe acute renal failure (serum creatinine >620 µmol/L).

In Germany, the hantavirus seroprevalence in humans is 1%–2%, but higher seroprevalences are found in disease-endemic regions, such as the Alb-Danube Region, South Germany (16). Most of our patients might have acquired PUUV infection during leisure activities, as occupations were not found to be risk factors in this study. Fluctuations of infected rodent populations may be responsible for the seasonal and endemic outbreaks of PUUV infections (4,6–18). Most patients with nephropathia epidemica resided to the north of the River Danube (Figure 1), which raises the possibility that the river acts as a geographic barrier for the migration of PUUV-infected rodent populations. However, direct evidence for this assumption was not provided in this study.

The diagnosis of an acute PUUV infection is established by detecting PUUV-specific IgM antibodies (19). Cross-reactive neutralizing antibodies to closely related hantavirus serotypes are common in the early but not in the convalescent phase of infection (20,21), as observed in two patients. In all patients, acute PUUV infection was proven by the different serologic tests, which were equally able to discriminate various hantavirus serotypes (Table 2).

Infections of endothelial and tubular cells, in conjunction with various immunologic mechanisms, are responsible for acute renal failure and thrombocytopenia in nephropathia epidemica. The maturation of hantavirus-infected dendritic cells may efficiently stimulate a pro-inflammatory immune response of specific T cells (22). β3 integrins are adhesive receptors on platelets and endothelial cells and may contribute to capillary leak and platelet activation in PUUV infection (3,23,24). Endothelial cell damage, circulating immune complexes, complement activation, T-cell activation, and cytokine response with high levels of tumor necrosis factor-α and interleukin-6 might cause acute renal failure and peripheral consumption of platelets (9,25,26). Similar to PUUV infections, severe thrombocytopenia may predict severe organ failure in other infectious diseases, including Rocky Mountain spotted fever (27).

In our patients, acute PUUV infection was a highly dynamic process, characterized by a short transient thrombocytopenia followed by mild-to-severe acute renal failure. We found statistical evidence that severe thrombocytopenia (<60 x 10^9^/L) is a significant early prognostic parameter for subsequent severe acute renal failure (serum creatinine >620 µmol/L). In other studies, thrombocytopenia was not identified as a predictive marker for severe acute renal failure (9,28); in these studies, laboratory data before hospital admission were not reported (9,28). These parameters were included to evaluate the earliest phase of infection. Leukocyte count was elevated in patients with a severe course of renal failure but was not predictive at first examination (Figure 3B). Similarly, quantitative hematuria, quantitative leukocyturia, and tubular cell casts obtained 1 day (range 0–12 days) after admission to hospital were associated with, but not predictive for, severe renal failure.

A specific therapy for nephropathia epidemica is not generally applicable, but a symptomatic therapy including hemodialysis may be individually required. If the platelet count is <60 x 10^9^/L in the early phase of infection, acute renal failure with serum creatinine >620 µmol/L is impending, and a tight control of clinical and laboratory parameters, including hospitalization, is mandatory. A platelet count >60 x 10^9^/L in the early phase of infection is predictive for mild renal failure, and outpatient treatment might be possible in absence of uremia, anuria, pulmonary symptoms, or other signs of severe infection. If a patient's platelet count has not dropped below 60 x 10^9^/L before day 6 after initial symptoms, the clinician may feel relatively comfortable predicting that severe renal failure will not occur. Later phases of infection are characterized by increased serum creatinine values but normalizing platelet counts (Figure 2). Early discharge from hospital may be justified in all patients at low risk for consecutive severe acute renal failure and in all patients with ongoing reconstitution of renal function. Thus, platelet counts obtained during the early phase of infection are suggested as a promising parameter for a risk-adapted, cost-effective disease management.
